# Effect of UV-B irradiated vitamin D enriched yeast supplementation on milk performance and blood chemical profiles in dairy cows

**DOI:** 10.5713/ab.23.0013

**Published:** 2023-05-04

**Authors:** Patipan Hnokaew, Tossapol Moonmanee, Chirawath Phatsara, Nattaphon Chongkasikit, Prayad Trirawong, Lukman Abiola Oluodo, Saowaluck Yammuen-Art

**Affiliations:** 1 Department of Animal and Aquatic Sciences, Faculty of Agriculture, Chiang Mai University, Chiang Mai 50200, Thailand; 2 Innovative Agriculture Research Center, Faculty of Agriculture, Chiang Mai University, Chiang Mai 50200, Thailand; 3 Department of Animal Science, Faculty of Agriculture at Kamphaeng Saen, Kasetsart University, Nakhon Pathom 73140, Thailand; 4 Outreach Department, Rubber Research Institute of Nigeria, Benin City, 1069, Nigeria

**Keywords:** Blood Chemical Profiles, Dairy Cows, UV-B Irradiated Vitamin D Enriched Yeast, Vitamin D in Milk

## Abstract

**Objective:**

The objective was to evaluate the effects of UV-B irradiated vitamin D-enriched yeast supplementation on milk yield, milk composition, vitamin D in milk, milk fatty acids, blood chemistry, and 25(OH)D status in dairy cows.

**Methods:**

Six Thai Friesian cows (milk production, 11.2±2.0 kg/d; body weight, 415.0±20.0 kg; and days in milk, 90.0±6.0) were allocated to each treatment in a 3×3 Latin square design, with three treatments and three periods. Each period of the Latin square lasted 49 days consisting of 14 days for diet adaptation and 35 days for sample collection. Dairy cows were randomly assigned to one of three treatments: i) feeding a basal diet without yeast (CON); ii) basal diet + 5 g of live yeast (75 IU/head/d of vitamin D_2_; LY); and iii) basal diet + 5 g of UV-B irradiated vitamin D enriched yeast (150,000 IU/head/d of vitamin D_2_; VDY). Feed intake and milk production were recorded daily, milk sample collection occurred on days 14 and 35 of each collection period, and blood plasma was collected on days 0, 7, 14, 21, 28, and 35 of each collection period.

**Results:**

The results show that after a trial period of 14 and 35 days, the VDY group had significantly higher vitamin D content in milk than the LY and CON groups (376.41 vs 305.15, 302.14 ng/L and 413.46 vs 306.76, 301.12 ng/L, respectively). At days 7, 14, 21, 28, and 35 of the experiment, cows fed the VDY group had significantly higher 25(OH)D_2_ status in blood than the CON and LY groups (51.07 vs 47.16, 48.05 ng/mL; 54.96 vs 45.43, 46.91 ng/mL; 56.16 vs 46.87, 47.16 ng/mL; 60.67 vs 44.39, 46.17 ng/mL and 63.91 vs 45.88, 46.88 ng/mL), respectively.

**Conclusion:**

In conclusion, UV-B irradiated vitamin D-enriched yeast supplementation could improve vitamin D content in the milk and 25(OH)D status in dairy cows during the lactation period.

## INTRODUCTION

Currently, the interest and attention in dairy production are aimed at improving its nutritional composition for increased product value and to serve consumer demands. Besides fats, proteins, and other substances, vitamin D is an important nutrient in dairy products. Vitamin D is not just essential for optimal skeletal growth and development; it also acts as a precursor to a complement endocrine process that maintains Ca and P concentrations in the blood [1–3]. Recently, vitamin D has been demonstrated to have numerous physiological functions, such as modulation of cellular differentiation and proliferation and activation of innate immunological responses [4–6]. Vitamin D has two types, including vitamin D_3_ (cholecalciferol), which is derived from animals, their skin can synthesize vitamin D_3_ by a photochemical conversion of its precursor (7-dehydrocholesterol), and vitamin D_2_ (ergocalciferol), which is produced by fungi and plants [7–9]. Importantly, vitamin D activation requires two steps of hydroxylation in the body. In the first step, vitamin D is converted in the liver to 25-hydroxyvitamin D (25(OH) D). The second step occurs predominantly in the kidneys and produces 1, 25-dihydroxyvitamin D (1, 25(OH)2D), which is physiologically active [10,11]. The content of vitamin D in milk is strongly influenced by the dietary ration composition of the dairy cows [12]. But dietary vitamin D interacts with the rumen’s microbial community before reaching the absorption site. The previous research indicated that a significant portion of vitamin D is degraded in the rumen into biologically inactive compounds [13,14]. *In vitro* research suggests that approximately 80% of vitamin D is broken down by bacteria during a 24-hour incubation in rumen fluid [13, 15,16], reducing its availability to these animals. In the same way, dairy cows receiving inadequate amounts of vitamin D cannot increase the vitamin D content of their milk [17]. If a method is developed to inhibit vitamin D from degrading in the rumen, the amount of vitamin D in dairy products may be increase. Yeast has significant levels of provitamin D_2_ (ergosterol), which is converted to vitamin D_2_ when exposed to UV light; therefore, supplementing with yeast may help improve undegradable vitamin D in the rumen. [18]. Absorption of ultraviolet-B radiation results in the maximum yield of vitamin D synthesis [19,20]. Moreover, yeast typically grows and survives in the rumen environment, which can enhance the production efficiency of ruminants [21,22]. Consequently, it can survive in the rumen without being destroyed by rumen microbes. Also, rumen microorganisms do not degrade vitamin D in yeast cells [16,23]. Vitamin D enriched yeast could prevent the loss of vitamin D that is typically degraded by rumen microorganisms. The remaining vitamin D content of UV-B irradiated vitamin D enriched yeast after 24 hours of *in vitro* incubation with total mixed ration revealed higher than the commercial vitamin D_2_ and vitamin D_3_ (93.76% vs 49.93% and 45.36%, respectively) [16]. However, the effects of UV-B irradiated vitamin D enriched yeast supplementation during lactation on milk products and blood chemistry in dairy cows are not well understood. Therefore, the current study aimed to evaluate the effects of UV-B irradiated vitamin D enriched yeast supplementation on milk yield, milk composition, vitamin D in milk, milk fatty acids, blood chemistry, and 25(OH)D concentration in dairy cows.

## MATERIALS AND METHODS

### Vitamin D enriched yeast production

Chiang Mai University Institutional Biosafety Committee has approved this protocol (CMUIBC0662003, Approval No. A0662002). *Baker’s yeast* was streaked on YM agar plates at 25°C for 48 hours, and single colony was picked into YM broth for 24 hours at 25°C. The enriched culture (1% v/v) was transferred to YM broth in a sterile Erlenmeyer flask at 25°C with 100 rpm rotation for 16 hours. The enriched culture (1% v/v) was transferred immediately in YM broth and incubated for 12 hours at 25°C with 100 rpm rotation. Following incubation, samples of yeast culture were exposed to UV-B irradiation for 10 hours in an irradiation box. Eight UV-B lamps (311±5 nm, Philips TL 20W/01 RS SLV/25) measuring 589.8 mm in length were placed 15 cm away from the sample to illuminate an area of 80×120 cm^2^. The UV-B irradiated yeasts were randomly collected 7 times out of a total of 21 times after UV-B irradiation for analysis of accuracy and precision of vitamin D enriched yeast production (before vitamin D enriched yeast were supplemented to the dairy cow diets). The yeast exposed UV-B was separated and evaluated for vitamin D concentration using procedures authorized by AOAC [24] and Mattila et al [25]. The seven random measurements of vitamin D enriched yeast products following UV-B irradiation range from 154,279.75 to 158,926.49 IU/5 g dry matter (DM), with an average of 156,650.18 IU/5 g DM.

### Animals, diet, and experimental design

This research was carried out in conformity with the international and national guidelines for the care and use of research animals. The Institutional Animal Care and Use Committee (Agricultural Animals) examined and approved all experimental protocols used in this study (AG01003/2565). Six Thai Friesian cows in the second lactation were grouped according to milk production (11.2±2.0 kg/d), body weight (BW; 415.0±20.0 kg), and days in milk (90.0±6.0), were used in a replicated 3×3 Latin square design, with three treatments and three periods. Each period had a 49-day duration, where the first 14 days of each period were for adaptation to the diets, and the last 35 days were for data and sample collection. They were randomly allocated to one of three treatments: i) basal diet without yeast (CON); ii) basal diet with 5 g of live yeast (LY; 75 IU/head/d of vitamin D_2_), and iii) basal diet with 5 g of UV-B irradiated vitamin D enriched yeast (VDY; 150,000 IU/head/d of vitamin D_2_). Specimens of yeast were mixed with a small amount of concentrate. They were fed daily to each cow in the experimental group before morning milking (each cow was fed a dietary additive separately in the milking stalls). All cows are housed in individual stall houses and have free access to drinking water and mineral lick blocks. The three treatments were fed ruzi grass *ad libitum* two times a day (08:00 and 16:00), and commercial concentrate (CO A NORTH company limited, Lumpun, Thailand) was fed 4.0 kg/head/d twice a day before milking (03:45; 2.0 kg and 14:45; 2.0 kg). The composition of ruzi grass and concentrate at the beginning of the study is presented in Table 1. The field trial was conducted from September 2021 to January 2022 at Mae Hai Farm, the Department of Animal and Aquatic Sciences, Faculty of Agriculture, Chiang Mai University, Chiang Mai, Thailand.

### Sampling

Feed intake was recorded daily, and feed samples were collected weekly for chemical analysis. All cows were milked twice daily at 04:00 and 15:00, and the milk yield was the sum of the two milking periods daily. Milk samples were collected on days 14 and 35 of each collection period. Two milk samples (morning and evening) were pooled (150 mL) according to yield and then split into 3×50 mL samples and immediately frozen at −20°C. Milk samples were analyzed for milk composition (fat, protein, lactose, total solid (TS), solid not fat (SNF), vitamin D concentration (vitamin D_2_, vitamin D_3_), and fatty acid profile.

Blood samples were collected from the jugular vein of each cow on days 0, 7, 14, 21, 28, and 35 of each collection period for plasma 25(OH)D_2_, 25(OH)D_3_, total calcium, phosphorus, blood urea nitrogen (BUN), alanine transaminase (ALT), alkaline phosphatase (ALP), total protein (TP), albumin and globulin analyses. Samples were collected in 4 mL vacutainers containing lithium heparin from each cow at each sampling time. Each collected sample was centrifuged immediately at 1,500×g for 15 min at 15°C to separate plasma, which was then stored at −20°C.

### Determination of milk composition

An automated milk analyzer examined the milk composition of samples (MilkoScan FT 2; FOSS, Hillerod, Denmark). According to the NRC [26], milk yields with 3.5% fat content and energy were calculated as follows:


(1) 3.5% fat-corrected milk (FCM)=0.4324×milk kg+(16.218×milk fat kg)

(2) Energy-corrected milk yield (ECM)=[(0.3246×milk yield)+(12.86×fat yield)+(7.04×protein yield)]

According to Folch et al [27], fatty acid composition of milk were analyzed. The lipids were extracted using 8 g milk samples and chloroform and methanol mixture (ratio 2:1 v/v; 100 mL). Fatty acid methyl esters (FAMEs) were prepared following the explanation of Morrison and Smith [28]. The Shimadzu GC-2030 gas chromatograph (Kyoto, Japan) was used to measure fatty acid profiles. The samples were isolated using a wall-coated fused wax capillary column (0.25 mm× 100 m×0.25 μm, RT-2560; RESTEK, Bellefonte, PA, USA). The injector’s temperatures were maintained at 250°C and the carrier gas used was helium. The oven temperature program was elevated at a rate of 10°C/min from 50°C to 220°C and kept for 35 min, then increased at a rate of 5°C/min from 200°C to 230°C and kept for 20 min. One μL of samples was injected, and the flame ionization detector temperature was fixed to 250°C. The samples were classified by comparing their peak retention time to those of the FAME mixture standard (RESTEK, USA) [29,30].

Vitamin D content in milk samples was extracted according to Gong and Ho [31]. The analytical column employed for the analysis of vitamin D_2_ was a reverse phase C18 column (5 μm, 4.6×250 mm; RESTEK, USA). The filtered samples were injected into an HPLC machine in 50 microliters (1220 Infinity II LC; Agilent Technologies, Santa Clara, CA, USA). The UV detection wavelength was set at 264 nm, and the mobile phase's composition was acetonitrile:methanol (75:25 v/v) at a flow rate of 1.3 mL/min. The amount of vitamin D was assessed by comparing the standards' retention times, and its quantification was calculated using a calibration curve.

### Measurement of blood biochemistry and 25(OH)D

Blood samples were analyzed using commercial kits at the Hematology and Biochemistry Lab, Small Animal Hospital, Faculty of Veterinary Medicine, Chiang Mai University, Chiang Mai, Thailand. The concentration of total calcium, phosphorus, BUN, ALT, ALP, TP, albumin, and globulin were analyzed on an automated clinical chemistry analyzer (Sysmex BX-3010; Sysmex Asia Pacific Pte Ltd, Chuo-ku, Japan).

The amounts of 25-hydroxyvitamin D in plasma samples were determined using HPLC technique. The plasma samples were mixed with acetonitrile (1:2 v/v of plasma:acetonitrile), and extracted according to Olkowski et al [32]. The present study used an analytical reverse phase C18 column (5 μm, 4.6×150 mm; Restek, USA). The injected volume was 50 μL. The mobile phase (100% acetonitrile) was delivered at a 2.0 mL/min flow rate. The UV detection was monitored at 264 nm. Calibration curves for measuring 25(OH)D in plasma were created using authentic standards of 25(OH)D_2_ and 25(OH)D_3_.

### Statistical analyses

Data from experiments were evaluated using the SPSS application. The IBM statistical package’s (20.0) results are displayed as mean±standard error mean. The effects of treatments on feed intake, milk production, milk composition, vitamin D in milk, fatty acid profile, and blood parameters were analyzed using a general linear model procedure for a Latin Square design. The model for analysis was: *γ*_ijk_ = *μ*+*ρ*_i_+*β*_j_+*τ*_k_+*e*_ijk_, Where *γ*_ijk_ = the observation of experiment; *μ* = overall mean; *ρ*_i_ = period effect; *β*_j_ = cow effect; *τ*_k_ = treatment effect; *e*_ijk_ = the random residual. The model included the fixed effects of treatment and period, and the random effect of cows nested within the squares. The Duncan’s multiple range test was selected for separation of treatment means. Statistical significance was defined as p<0.05.

## RESULTS

### Lactation performance

Dry matter intake was not influenced by live yeast and UV-B irradiated vitamin D enriched yeast treatments throughout the experiment (Table 2). After the first 14 days, dairy cows from the LY and VDY groups had milk lactose levels higher than the CON group (4.37%, 4.38% vs 4.28%; p = 0.007), while the VDY group had a higher vitamin D_2_ content than the CON and LY groups (376.41 vs 305.15, 302.14 ng/L; p<0.001). After a trial period of 35 days, the LY and VDY groups had higher milk lactose levels than the CON group (4.33%, 4.34% vs 4.23%; p = 0.010), compared to the dairy cows in the CON and LY groups, those treated with VDY produced more vitamin D_2_ content (413.46 vs 306.76, 301.12 ng/L; p<0.001). There was no significant effect of treatment on milk yield, FCM 3.5%, ECM, fat yield, protein yield, milk fat, milk protein, TS, SNF, and vitamin D_3_ content in milk throughout the first 14 days and 35 days of the study, as is shown in Table 2.

### Milk fatty acid profile

The concentrations of fatty acid composition in milk after the 35-day experimental feeding are displayed in Table 3. Dietary supplements had no effect on the milk fatty acid profile except for a few minor changes compared to the CON group; oleic acid (C18:1) tends to be higher in the LY and VDY groups (29.83, 29.99, vs 29.00 g/100 g; p = 0.051). The treatment had no effect on the other fatty acids.

### Blood biochemistry and vitamin D metabolites

**Figure 1:**
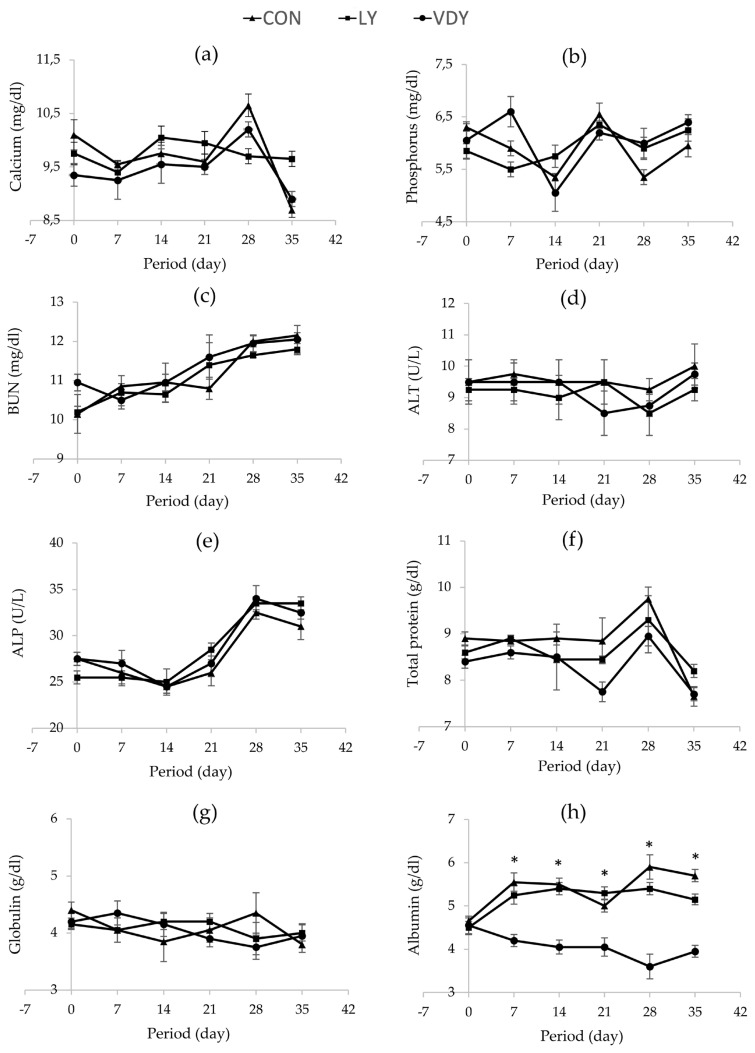
Effect of treatments on blood chemistry concentrations in plasma. Treatment: i) feeding a basal diet without yeast (CON); ii) basal diet + 5 g of live yeast (75 IU/head/d of vitamin D_2_; LY); and iii) basal diet + 5 g of UV-B irradiated vitamin D enriched yeast (150,000 IU/head/d of vitamin D_2_; VDY). ALP, alkaline phosphatase; ALT AAlanine transaminase; BUN, blood urea nitrogen. * Significantly different at p<0.05.

**Figure 2:**
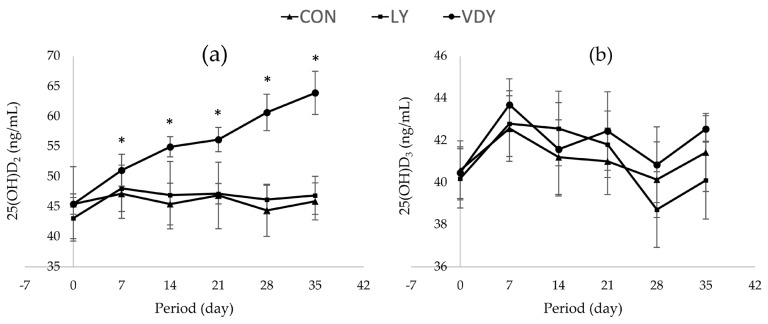
Effect of treatments on 25-hrdroxyvitamin D [25(OH)D] concentrations in plasma. Treatment: i) basal diet without yeast (CON); ii) basal diet + 5 g of live yeast (75 IU/head/d of vitamin D_2_; LY); and iii) basal diet + 5 g of UV-B irradiated vitamin D enriched yeast (150,000 IU/head/d of vitamin D_2_; VDY). * Significantly different at p<0.05.

Figure 1 shows the concentrations of Ca and P in blood plasma, renal and hepatic metabolites throughout the experiment. The treatments did not affect concentrations of calcium (Figure 1a), phosphorus (Figure 1b), BUN (Figure 1c), ALT (Figure 1d), ALP (Figure 1e), TP (Figure 1f), and globulin (Figure 1g) in plasma. Cows supplemented with VDY group had lower albumin than CON and LY groups by days 7 (3.60 vs 5.90, 5.40 g/dL, p = 0.012), days 14 (4.05 vs 5.50, 5.40 g/dL; p = 0.002), days 21 (4.05 vs 5.00, 5.30 g/dL; p = 0.010), days 28 (4.05 vs 5.50, 5.40 g/dL; p = 0.005) and days 35 (3.95 vs 5.70, 5.15 g/dL; p = 0.001), respectively. Additionally, a significantly higher concentration of 25-hydroxyvitamin D_2_ was observed in the cows fed the VDY group than the CON and LY groups by days 7 (51.07 vs 47.16, 48.05 ng/mL; p = 0.009), days 14 (54.96 vs 45.43, 46.91 ng/mL; p<0.001), days 21 (56.16 vs 46.87, 47.16 ng/mL; p<0.001), days 28 (60.67 vs 44.39, 46.17 ng/mL; p<0.001) and days 35 (63.91 vs 45.88, 46.88 ng/mL; p<0.001), respectively (Figure 2a). Furthermore, the 25-hydroxyvitamin D_3_ concentration was not affected by treatment (Figure 2b).

## DISCUSSION

Milk lactose was higher in the milk of dairy cows treated with the LY and VDY groups compared to the CON group. This could be the influence of yeast which is an effective source of probiotics that can stimulate microbial activity in the rumen [33]. Specifically, it promotes rapid growth and activity of rumen-lactate-utilizing bacteria, such as *Selenomonas ruminantium* and *Megasphera elsdenii* [34]. *M. elsdenii* is the most predominant species because it can utilize lactic acid in the rumen. It is also responsible for up to 80% of all lactic acid fermentation to propionic acid, and *S. ruminantium* can convert lactic acid into propionic acid [35–38]. Monteiro et al [37] reported that adding live yeast increased propionate concentration in the rumen (38.8% vs 31.8% of total volatile fatty acid). Similarly, Hnokaew and Yammuan-art [16] observed that UV-B irradiated vitamin D enriched yeast and live yeast supplementation in corn silage produced higher propionate than control groups after 24 hours of *in vitro* incubation (10.61, 10.43, vs 5.29 mmol, respectively). Propionates are converted into glucose and allowed to participate in the production of lactose, one glucose and one galactose molecule conjoin with 1, 4 - galactoside bonds to form the two molecules of sugar known as lactose that can be found in milk. Up to 80% of the glucose needed for this synthesis comes from the circulation, which is absorbed from the rumen. Osmotic pressure was then used to further move lactose through the cells and into the alveoli [39–41]. Usually, the quantity of vitamin D received through dietary supplementation affects the amount of vitamin D found in dairy cow milk. McDermott et al [17] reported that supplementing cow diets with a higher daily dose of vitamin D_3_ (250,000 IU/d) increased vitamin D_3_ concentration in milk from 75 to 325 ng/L. Hollis et al [42] fed 400,000 IU of vitamin D_3_ daily to dairy cows and discovered that vitamin D_3_ concentration in milk increased from 43 to 322 ng/L. In the present study, dairy cows received vitamin D-enriched yeast, averaging 156,650.18 IU/head/d. Also, yeast may thrive in anaerobic environments (in the rumen condition). To obtain energy for growth, yeast can use glucose and oligosaccharides formed by the digestion of amylolytic bacteria [23]. The fact that they can survive in the rumen without being broken down by microorganisms is crucial. As a result, rumen bacteria do not break down vitamin D in yeast cells [16]. Vitamin D can be absorbed and utilized more efficiently after entering the small intestine, leading to an increased vitamin D concentration in milk, according to Figure 2a.

The UV-B irradiated vitamin D enriched yeast and live yeast in diets had no influence on the fatty acid profiles in milk. Each group received the same basal diet, differing only in yeast supplementation. Live yeast cells do not contain a significant amount of lipids; thus, the possible effects on milk fat composition could be predicted to implicate changes in the relative abundance of odd- and branched-chain fatty acid and certain biohydrogenation intermediates from the rumen. Research by Bayat et al [43] reported that fatty acid composition in milk cows with or without live yeast had no significant influence on ruminal lipolysis, biohydrogenation, or microbial lipid synthesis. According to Longuski et al [44] and Yalcin et al [45] reported that live yeast culture supplementation had no effect on milk fatty acids except for oleic acid (C_18:1_), while the LY and VDY groups tend to be more than the CON group in this study. The influence of live yeast supplementation on ruminal biohydrogenation processes (BH) was initially discovered by Julien et al [34]. So, LY could therefore have an impact on several stages of BH, starting with the biohydrogenating microorganisms by promoting the growth of bacteria that produce the t11 or t10 isomer. Secondly, by altering the ruminal biotope, i.e., by regulating ruminal pH or maintaining stable rumen environment. Furthermore, supplementing with live yeast boosted C_18:1_ accumulation and reduced the C_18:0_ ratio [46, 47]. Therefore, it may be hypothesized that the improved rumen conditions caused by LY administration favored the production of intermediate fatty acids in the rumen, which led to the production of C_18:1_.

Supplementing cows with UV-B irradiated vitamin D enriched yeast decreased albumin in blood plasma because it is a specific protein that binds to vitamin D and serves to transport vitamin D into the bloodstream. Vitamin D synthesized in the skin or absorbed in small intestine, was released into the blood and bound to vitamin D-binding protein (DBP), which serves as its primary transport medium in the circulation of vitamin D [48]. DBP is structurally related to albumin and binds to all naturally occurring albumin in the blood [49]. Once in the bloodstream, vitamin D is attached to its serum carrier protein, DBP, and albumin [50,51]. After hydroxylation by 25-hydroxylase in the liver [52], it again binds to either DBP or albumin for endocrine transport to target tissues [53]. The majority of circulating 25(OH) D is tightly bound to DBP, with the leftover bound to albumin [54,55]. As a result, dairy cows fed UV-B irradiated vitamin D-enriched yeast had lower albumin levels than those without supplementation. The 25(OH)D_2_ concentration increased in the blood because dairy cows received enhanced vitamin D from vitamin D-enriched yeast, and UV-B irradiated vitamin D-enriched yeast which can also prevent degradation from microorganisms in the rumen, making it more usable for dairy cows. According to a prior study, cows given vitamin D_3_ supplements of 250,000 IU/d had higher average levels of 25(OH)D_3_ than the control group (70 vs 30 ng/mL) [17]. Numerous investigations discovered that supplementing dairy cows with yeast culture had no effect on BUN, ALT, ALP, TP, or globulin levels [45, 56,57].

## CONCLUSION

In this study, feeding dairy cows with 150,000 IU/head/d of UV-B irradiated vitamin D enriched yeast improved vitamin D_2_ content in milk and 25(OH)D_2_ status in blood during the lactation period without detrimental effects on the health status and overall milk production. In the same way, UV-B irradiated vitamin D enriched yeast supplementation tended to improve milk lactose and palmitoleic acid (C18:1) in milk. Further research should be focused on the influence of vitamin D enriched yeast levels on reproductive performance, and the immune responses in lactating dairy cows.

## Figures and Tables

**Table 1:** Dietary nutrient composition and vitamin D content of ruzi grass and concentrate

Item	Ingredient	

	Ruzi	Concentrate
Chemical composition (DM basis, %)		
Dry matter	20.24	92.14
Organic matter	90.08	90.49
Crude protein	7.42	20.55
Ether extract	3.63	5.32
NDF	72.12	43.11
ADF	43.11	19.78
ADL	8.66	7.78
Hemicellulose	29.09	32.40
Cellulose	34.44	11.99
Vitamin D content (IU/kg DM)		
Vitamin D_2_	452.80	84.31
Vitamin D_3_	ND	ND

DM, dry matter; NDF, neutral detergent fiber; ADF, acid detergent fiber; ADL, acid detergent lignin, ND, not detected.

**Table 2:** Milk production response to live yeast culture (LY) and UV-B irradiated vitamin D enriched yeast (VDY)

Item	Treatment^1)^			SEM	p-value

	CON	LY	VDY		
	------------------- Day 14 -------------				
DMI (kg/d)					
Ruzi grass	8.68	9.12	8.95	0.62	0.329
Concentrate	3.69	3.69	3.69	-	-
Yield (kg/d)					
Milk	11.18	11.25	11.51	0.35	0.481
3.5% FCM	10.36	10.65	11.01	0.33	0.395
ECM	10.59	10.73	11.11	0.32	0.459
Fat	0.34	0.36	0.37	0.01	0.458
Protein	0.34	0.33	0.35	0.01	0.414
Milk composition (%)					
Fat	3.11	3.19	3.26	0.10	0.741
Protein	3.04	3.10	3.14	0.04	0.353
Lactose	4.28^A^	4.37^B^	4.38^B^	0.02	0.007
Total solid	11.17	11.44	10.82	0.26	0.605
SNF	8.28	8.25	8.26	0.04	0.137
Vitamin D (ng/L)					
D_2_	305.15^A^	302.14^A^	376.41^B^	6.054	<0.001
D_3_	269.40	277.59	277.87	2.513	0.232
	------------------ Day 35 ---------------				
DMI (kg/d)					
Ruzi grass	9.06	8.73	9.20	0.65	0.382
Concentrate	3.69	3.69	3.69	-	-
Yield (kg/d)					
Milk	10.75	10.76	10.62	0.37	0.907
3.5% FCM	10.39	10.41	10.46	0.30	0.984
ECM	10.46	10.45	10.52	0.28	0.980
Fat	0.35	0.36	0.36	0.01	0.916
Protein	0.33	0.32	0.33	0.01	0.932
Milk composition (%)					
Fat	3.36	3.37	3.46	0.10	0.782
Protein	3.06	3.07	3.12	0.04	0.253
Lactose	4.23^A^	4.33^B^	4.34^B^	0.03	0.010
Total solid	11.72	11.54	11.67	0.11	0.565
SNF	8.17	8.14	8.15	0.04	0.200
Vitamin D (ng/L)					
D_2_	306.76^A^	301.12^A^	413.46^B^	7.490	<0.001
D_3_	266.01	265.80	274.32	3.729	0.164

SEM, standard error of the mean; DMI, dry matter intake; FCM, fat-corrected milk; ECM, energy-corrected milk; SNF, solid not fat.

1) Treatment were i) feeding a basal diet without yeast (CON); ii) basal diet + 5 g of live yeast (75 IU/head/day of vitamin D_2_; LY); and iii) basal diet + 5 g of UV-B irradiated vitamin D enriched yeast (150,000 IU/head/d of vitamin D_2_; VDY).

A,B Mean values within rows with different superscripts were significantly different at p<0.05.

**Table 3:** Milk fatty acid composition (g/100 g) response to live yeast culture (LY) and UV-B irradiated vitamin D enriched yeast (VDY) after 35 days of experimental feeding

Fatty acid	Treatment^1)^			SEM	p-value

	CON	LY	VDY		
C_4:0_	1.66	1.61	1.65	0.05	0.690
C_6:0_	1.42	1.55	1.45	0.07	0.761
C_8:0_	0.74	0.81	0.78	0.02	0.200
C_10:0_	1.45	1.55	1.48	0.02	0.299
C_11:0_	0.19	0.19	0.19	0.01	0.903
C_12:0_	2.22	2.18	2.20	0.05	0.217
C_13:0_	0.10	0.11	0.11	0.02	0.140
C_14:0_	9.04	8.41	8.24	0.19	0.220
C_14:1_	0.96	1.13	1.18	0.07	0.497
C_15:0_	1.33	1.25	1.21	0.09	0.865
C_16:0_	30.09	29.91	30.01	0.26	0.940
C_16:1_	2.28	2.30	2.32	0.08	0.976
C_17:0_	0.74	0.75	0.71	0.01	0.221
C_17:1_	0.32	0.30	0.31	0.01	0.728
C_18:0_	13.27	12.90	13.46	0.24	0.576
C_18:1_	29.00	29.83	29.99	0.14	0.051
C_18:26t_	0.29	0.32	0.28	0.01	0.279
C_18:26c_	1.68	1.62	1.64	0.02	0.154
C_20:0_	0.29	0.28	0.26	0.01	0.272
C_21:0_	1.16	1.17	1.06	0.06	0.757
C_22:0_	0.15	0.14	0.13	0.01	0.671
C_23:0_	0.13	0.12	0.12	0.01	0.313
C_24:0_	0.08	0.08	0.07	0.01	0.091
∑SFA	63.87	63.01	63.14	0.23	0.587
∑MUFA	32.67	32.72	33.79	0.20	0.094
∑PUFA	1.97	1.94	1.93	0.02	0.440

SEM, standard error of the mean; SFA, saturated fatty acids; MUFA, monounsaturated fatty acids; PUFA, polyunsaturated fatty acids.

1) Treatment were i) feeding a basal diet without yeast (CON); ii) basal diet + 5 g of live yeast (75 IU/head/d of vitamin D_2_; LY), and iii) basal diet + 5 g of UV-B irradiated vitamin D enriched yeast (150,000 IU/head/d of vitamin D_2_; VDY).
